# Robot-assisted Kidney Transplantation from Deceased Donor: The European Experience

**DOI:** 10.1016/j.euros.2025.06.013

**Published:** 2025-08-06

**Authors:** Thomas Prudhomme, Lluis Peri, Alessio Pecoraro, Angelo Territo, Begoña Etcheverry, Milla Ortved, Andreas Røder, Idu Mirza, Graziano Vignolini, Malene Rohrsted, Byron López de Mesa Rodriguez, Jeremy Mercier, Nicolas Doumerc, Lorenzo Masieri, Francesc Vigues, Alberto Breda, Sergio Serni, Antonio Alcaraz, Mireia Musquera, Riccardo Campi

**Affiliations:** a Department of Urology, Hospital Clinic, Barcelona, Spain; b Department of Urology, Rangueil University Hospital, Toulouse, France; c Department of Experimental and Clinical Medicine, University of Florence, Florence, Italy; d Unit of Urology and Renal Transplantation, Oncology Department, Careggi University Hospital, Florence, Italy; e Department of Urology, Fundació Puigvert, Universitat Autonoma de Barcelona, Barcelona, Spain; f Department of Urology, Hospital Universitari de Bellvitge, L’Hospitalet de Llobregat, Barcelona, Spain; g Department of Urology, Centre for Cancer and Organ Diseases, Copenhagen University Hospital - Rigshospitalet, Copenhagen, Denmark

**Keywords:** Robotic-assisted kidney transplantation, Deceased donor, Delayed graft function, Surgical complications, Kidney transplantation

## Abstract

**Background and objective:**

In the context of deceased donors (DDs), robotic-assisted kidney transplantation (RAKT) is underutilized due to specific logistical and technical challenges. The aim of this study was to report the outcomes of DD-RAKT performed in centers involved in the European Association of Urology Robotic Urology Section (ERUS)-RAKT working group.

**Methods:**

This retrospective analysis is based on a prospectively collected multicenter database including data on DD-RAKT performed at seven referral ERUS-RAKT European centers from July 2015 to April 2024. Intraoperative, perioperative, and midterm functional outcomes after DD-RAKT were assessed. The decision-making strategy regarding the selection of a robotic versus an open surgical approach for kidney transplantation was also reported across the included centers.

**Key findings and limitations:**

A total of 67 patients with a median age of 36 yr (interquartile range [IQR]: 24–58) underwent DD-RAKT. Most donors were donors after brain death (80.6%), followed by donors after uncontrolled circulatory death (10.4%); there were 26 “expanded criteria” donors (39%). The median cold ischemia time was 14.8 h (IQR: 11.0–19.0). Eight patients (11.9%) had orthotopic DD-RAKT, while 59 patients (88.1%) had heterotopic DD-RAKT. The median operative time and the median rewarming time were 220 min (IQR: 180.0–252.0) and 50 min (IQR: 42–60), respectively. The major postoperative surgical complications (Clavien-Dindo ≥3) occurred in 11 patients (16%). Eighteen patients (27%) experienced delayed graft function. At a median follow-up of 27.1 mo (IQR: 9.5–59.4), graft survival rate was 92.6% and patient survival rate was 97.0%. The last median estimated glomerular filtration rate was 55.0 ml/min/1.73 m^2^ (IQR: 41.5–70.0). The main study limitations are its retrospective nature and the lack of a comparator group.

**Conclusions and clinical implications:**

DD-RAKT is feasible and safe at experienced centers, providing a viable way of expanding transplantation access to patients with end-stage kidney disease. Notwithstanding the logistical barriers for DD-RAKT, careful recipient selection and organ matching are key factors to achieve the best perioperative and functional outcomes.

**Patient summary:**

Robotic kidney transplantation using grafts from deceased donors is technically feasible and safe in well-selected recipients at experienced referral centers, showing favorable intra- and postoperative outcomes. While robotic kidney transplantation from deceased donors may be challenging to implement in clinical practice due to organizational and logistical barriers (which may vary across hospitals and countries), careful recipient selection is essential to ensure the best perioperative and functional outcomes.

## Take Home Message

In the context of deceased donors, robotic-assisted kidney transplantation presents satisfactory results and provides a viable way of expanding transplantation access to patients with end-stage renal disease considered at risk of open kidney transplantation. A careful selection of potential recipients is of utmost importance.

## 1 Introduction

Kidney transplantation (KT) is the treatment of choice for patients with end-stage kidney disease (ESKD) providing the best survival outcomes and quality of life [1,2]. Since the initial successful case in 1954, conventional open kidney transplant (OKT) surgery is still the gold standard procedure [3]. However, over the last 10 yr, elective robot-assisted kidney transplantation (RAKT) from living donors has been shown to achieve favorable outcomes [[4], [5], [6], [7], [8], [9], [10], [11]] and have the potential to minimize surgical morbidity as compared with OKT [12,13]. Nonetheless, in the context of deceased donors (DDs), RAKT is still controversial and underutilized due to specific logistical challenges, while this type of donor represents the majority throughout the world [14,15]. RAKT is performed only by surgeons with extensive experience in robotic and KT surgeries. Unlike living donor KT, which is performed as part of elective surgery, KT from a DD is performed in an emergency situation. Thus, in order to perform RAKT from DDs (DD-RAKT), both surgeons and robotic platforms have to be available.

In this specific frail population, expanding the indications for DD-RAKT is of utmost importance so as not to deprive recipients without living donors of the benefits of minimally invasive surgery.

Few studies evaluated the feasibility and safety of DD-RAKT. Recently, the Careggi Hospital team in Florence reported their experience on RAKT from donors after brain death (DBDs) [[16], [17], [18]], in comparison with conventional OKT, as well as the group from the University of Illinois at Chicago [19]. However, to date, no multicentric study evaluated the effectiveness of DD-RAKT.

To fill this gap, we reported the intraoperative, perioperative, and functional outcomes of DD-RAKT performed in centers involved in the European Association of Urology Robotic Urology Section (ERUS)-RAKT working group.

## 2 Patients and methods

### 2.1 Patients and database

The ERUS-RAKT project has been described previously [5,7]. After ethical committee approval, data from consecutive patients undergoing RAKT were prospectively collected into the multi-institutional ERUS-RAKT common database. For this study, we reviewed the database to select DD-RAKT cases performed between July 1, 2015 and April 30, 2024. DDs included the following types: (1) DBDs, (2) donors after controlled circulatory death (cDCDs; Maastricht III), and (3) donors after uncontrolled circulatory death (uDCDs; Maastricht II). DBDs were considered “expanded criteria donors” if they were aged >60 or 50–59 yr with two of the following features: history of hypertension, terminal serum creatinine ≥1.5 mg/dl, or death resulting from a cerebrovascular accident [14]. Patients who underwent RAKT from living donors were excluded from this study.

Cold ischemia time was defined as the duration of cold storage, while rewarming time was defined as the time between graft insertion in the abdominal cavity and revascularization. The estimated glomerular filtration rate (eGFR) was calculated using the Chronic Kidney Disease Epidemiology Collaboration formula [20]. Delayed graft function (DGF) was defined as the need of dialysis in the 1st operation week [14]. Intraoperative complications were reported according to the Intraoperative Adverse Incident Classification (EAUiaiC) by the European Association of Urology (EAU) Ad Hoc Complications Guidelines Panel [21], while postoperative surgical complications were reported according to the modified Clavien-Dindo system [22] and high-grade postoperative complications were defined as Clavien-Dindo grade ≥3. *Trifecta* was defined as the contemporary achievement of the following outcomes: (1) no DGF, (2) no major (Clavien-Dindo grade ≥3) postoperative surgical complications, and (3) eGFR ≥30 ml/min/1.73 m^2^ at hospital discharge. Computed tomography angiogram was performed for all recipients to assess their vascular anatomy and the potential presence of atherosclerotic plaques of iliac vessels.

### 2.2 Decision-making strategies regarding selection of robotic surgical approach within the ERUS-RAKT group

**Fig. 1:**
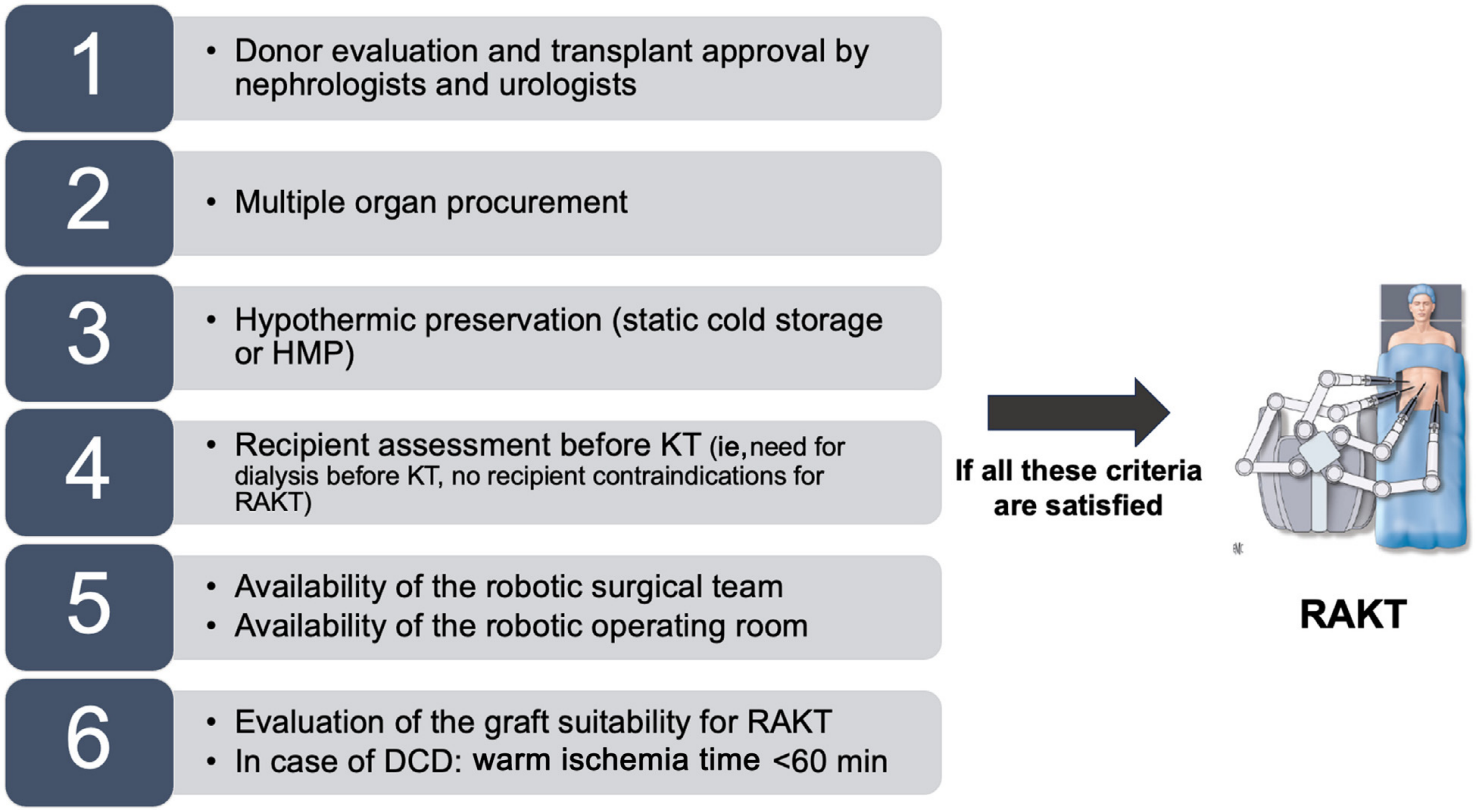
Decision-making process for the selection of RAKT candidates from deceased donors within the ERUS-RAKT group. First, a careful evaluation of the donor is performed and the transplant is approved by the nephrologists and urologists (1). Then, multiple organ procurement is performed (2) and the transplant is preserved in hypothermic conditions (SCS or HMP) (3). Next, the recipient is admitted to the hospital and a preoperative assessment is performed (ie, need for dialysis before KT and assessment of the absence of contraindication to RAKT) (4). Then, the availability of the robotic surgical team and the robotic operating room is checked (5). During bench surgery, graft suitability for RAKT is assessed focusing on vascular anomalies. In the specific case of DCDs, warm ischemia time should be <60 min (6). DCD = donor after circulatory death; ERUS = European Association of Urology Robotic Urology Section; HMP = hypothermic machine perfusion; KT = kidney transplantation; RAKT = robotic-assisted kidney transplantation; SCS = static cold storage.

A decision-making strategy regarding the selection of the robotic surgical approach has been reported within the different centers (Fig. 1). This decision-making strategy has been implemented according to the experience of the University of Florence [[16], [17], [18]].

First, a careful evaluation of the donor is performed and the transplant kidney is approved by the nephrologists and urologists. Then, the multiple organ procurement is performed and the transplant is preserved in hypothermic conditions (static cold storage or hypothermic machine perfusion). The recipient is admitted to the hospital, and a preoperative assessment is performed (ie, need for dialysis before KT, assessment of the absence of contraindication to RAKT including previous major abdominal surgeries, and severe atherosclerotic plaques at the level of iliac vessels). Then, the availability of the robotic surgical team (surgeon on call and nurses) and the robotic operating room is checked. During bench surgery, graft suitability for RAKT is assessed focusing on vascular anomalies. In the specific case of donors after circulatory death (DCDs), a warm ischemia time of <60 min was selected. All DD-RAKT procedures were performed by highly experienced robotic and transplant surgeons. DD-RAKT was performed only if the RAKT surgeon was on charge and all the above-mentioned criteria for RAKT were met.

### 2.3 Surgical technique

A detailed step-by-step description of the surgical technique for heterotopic DD-RAKT has been reported by the Florence team [[16], [17], [18]] and followed the principles of the Vattikuti-Medanta technique, using a transperitoneal approach [4,23]. Briefly, during bench surgery, the graft is prepared by the transplant surgeon and placed in a gauze jacket. In case of multiples arteries, the surgeon may use different techniques in order to reconstruct the renal artery or decide to perform separate arterial anastomoses during the robotic procedure. The surgical procedure was performed using either the da Vinci Si or the Xi robotic platform (Intuitive Surgical Inc., Sunnyvale, CA, USA) in a four-arm configuration, with a 0° lens and a 25° Trendelenburg tilt. Pneumoperitoneum was set at 8–12 mmHg and maintained constant through the use of the Airseal system. The graft was introduced through a Pfannenstiel or midline incision using a GelPoint device or the Alexis system. Vascular anastomoses were completed in an end-to-side fashion to the external iliac vessels using a 5-0 or 6-0 GORE-TEX suture (Gore Medical, Flagstaff, AZ, USA). Graft reperfusion was assessed by intraoperative Doppler ultrasound or using intraoperative FireFly fluorescence vascular imaging with indocyanine green. A ureterovesical anastomosis is then completed according to the modified Lich-Gregoire technique.

The surgical technique for orthotopic DD-RAKT has been reported recently by Vigues et al [24,25]. The procedure was performed via a transperitoneal approach, with the patient placed in the right lateral decubitus position. Briefly, after trocar placement, the splenic artery was first dissected and clamped using a bulldog clamp. Then, a robotic left nephrectomy is performed (with vascular clamping using bulldog clamps). After similar bench surgery, the graft is then introduced into the abdominal cavity and maintained in the ideal position by the gauze for subsequent vascular anastomoses using the fourth arm. Vascular anastomoses were completed in an end-to-end anastomosis between the graft vein and the left renal vein, and in an end-to-end anastomosis between the graft artery and the splenic artery, using a 6/0 Gore-Tex for both anastomoses. Urinary reconstruction was achieved via either a side-to-side ureteroureteral anastomosis or an end-to-end pyeloureteral anastomosis.

### 2.4 Statistical analysis

Descriptive statistics were calculated, with the median and interquartile range (IQR) reported for continuous variables, and the frequency and proportion reported for categorical variables. Differences between subgroups for donor kidney status (DBD vs cDCD vs uDCD) were evaluated using the Pearson χ^2^ and Kruskal-Wallis tests. Patient and graft survival were estimated using the Kaplan-Meier method. A *p* value of <0.05 was considered statistically significant. Statistical analyses were performed using S Prism 10.1.1 (GraphPad Software Inc, La Jolla, CA, USA) and SPSS v.27 (IBM, Armonk, NY, USA).

## 3 Results

### 3.1 Preoperative characteristics and kidney procurement outcomes

**Fig. 2:**
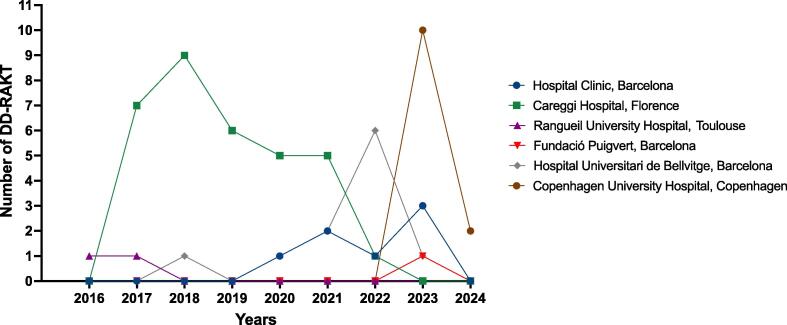
Number of deceased donor RAKT procedures performed by each center by year. DD-RAKT = deceased donor robotic-assisted kidney transplantation.

Overall, 67 patients were included in the analytic cohort. Of these patients, 41.2% (7/17) of ERUS-RAKT working group centers underwent DD-RAKT. The number of cases performed by each center during the study period is shown in Fig. 2. Preoperative donors, recipients, graft characteristics, and kidney procurement outcomes are reported in Table 1.

**Table 1:** Preoperative donor, recipient, and graft characteristics and kidney procurement outcomes

		Overall population (*n* = 67)	DBD (*n* = 54)	cDCD (*n* = 6)	uDCD (*n* = 7)	*p* value
*Donor characteristics*						
Cause of death, *n* (%)	Stroke	28 (41.8)	25 (46.3)	3 (50)	0 (0)	**<0.0001**
	Trauma	6 (9.0)	5 (9.3)	1 (16.7)	0 (0)	
	Cardiac arrest	8 (11.9)	0 (0)	1 (16.7)	7 (100)	
	Other	22 (32.8)	22 (40.7)	0 (0)	0 (0)	
	Unknown	3 (4.5)	2 (3.7)	1 (16.7)	0 (0)	
Age (yr), median (IQR)		54.0 (39.5–62.5)	52.0 (42.3–61.0)	73.0 (25.0–76.8)	43.0 (35.0–57.0)	0.3
BMI (kg/m^2^), median (IQR)		24.0 (22.0–29.0)	24.0 (22.0–29.0)	24.0 (22.0–25.0)	27.0 (23.0–30.0)	0.8
Male, *n* (%)		33 (49.3)	26 (48.1)	2 (33.3)	5 (71.4)	0.4
Expanded criteria donor, *n* (%)		26 (38.8)	13 (24.1)	6 (100)	7 (100)	**<0.0001**
eGFR (ml/min/1.73 m^2^), median (IQR)		90.0 (65.9–90.2)	90.0 (72.0–98.0)	90.0 (61.8–91.0)	78.0 (54.2–90.0)	0.4
*Recipient characteristics*						
Age (yr), median (IQR)		36.0 (24.0–58.0)	37.0 (23.8–58.3)	51.0 (27.3–66.5)	24.0 (22.0–38.0)	0.1
Male, *n* (%)		43 (64.2)	33 (61.1)	5 (83.3)	5 (71.4)	0.6
BMI (kg/m^2^), median (IQR)		24.8 (23.0–27.8)	25.0 (22.9–28.0)	25.0 (22.5–26.4)	24.0 (22.0–27.0)	0.8
Charlson Comorbidity Index >2, *n* (%)		26 (38.8)	20 (37.0)	2 (33.3)	4 (57.1)	0.6
Recipient nephropathy, *n* (%)	Autosomal dominant polycystic kidney disease	11 (16.4)	10 (18.5)	1 (12.5)	0 (0)	
	IGA nephropathy	7 (10.4)	7 (13.0)	0 (0)	0 (0)	0.8
	Vascular nephropathy	4 (6.0)	3 (5.6)	0 (0)	1 (14.3)	
	Diabetic nephropathy	5 (7.5)	4 (7.4)	1 (12.5)	0 (0)	
	Membranous glomerulonephritis	2 (3.0)	2 (3.7)	0 (0)	0 (0)	
	Postinfectious GN	4 (6.0)	4 (7.4)	0 (0)	0 (0)	
	Congenital nephropathy	3 (4.5)	2	2 (25.0)	1 (14.3)	
	Focal segmental glomerulosclerosis	1 (1.5)	1 (1.9)	0 (0)	0	
	Hypertensive nephropathy	5 (7.5)	4 (7.4)	0 (0)	1 (14.3)	
	Lupus nephritis	1 (1.5)	1 (1.9)	0 (0)	0	
	Schonlein-Henoch purpura	1 (1.5)	1 (1.9)	0 (0)	0	
	Tubolointerstitial nephritis	3 (4.5)	3 (5.6)	0 (0)	0	
	Other/unknown	20 (29.9)	12 (22.2)	4 (50.0)	4 (57.1)	
Native nephrectomy, *n* (%)		7 (10.4)	5 (9.3)	2 (33.3)	0 (0)	0.1
Major previous abdominal surgery, *n* (%)		20 (29.9)	14 (25.9)	2 (33.3)	4 (57.1)	0.2
Previous kidney transplantation, *n* (%)		5 (7.5)	4 (7.4)	1 (16.7)	0 (0)	0.5
Antiplatelet/anticoagulant therapy at surgery, *n* (%)	Antiplatelet	10 (6.7)	8 (14.8)	0 (0)	2 (28.6)	0.4
	Anticoagulant	2 (3.0)	2 (3.7)	0 (0)	0 (0)	0.8
Preemptive recipient, *n* (%)		17 (25.4)	14 (25.9)	0 (0)	3 (42.9)	0.2
Type of dialysis (if not pre-emptive), *n* (%)	Peritoneal dialysis	15 (22.4)	12 (22.2)	3 (50.0)	0 (0)	0.1
	Hemodialysis	35 (52.2)	28 (51.9)	3 (50.0)	4 (57.1)	0.9
Time on dialysis (mo), median (IQR)		24.0 (13.5–47.0)	24.0 (13.3–49.5)	21.0 (6.8–30.9)	41.0 (27.0–137.5)	0.2
*Graft characteristics*						
Right-sided graft, *n* (%)		37 (55.2)	31 (57.4)	4 (66.7)	2 (28.6)	0.3
Biopsy of the graft on the back table, *n* (%)		36 (53.7)	25 (46.3)	4 (66.7)	7 (100)	**0.02**
Karpinsky score (at biopsy), median (IQR)		3.0 (2.0–4.0)	3.0 (2.0–4.0)	2.0 (1.3–3.5)	2.0 (2.0–3.5)–	0.6
Graft with multiple arteries, *n* (%)		12 (17.9)	8 (14.8)	1 (16.7)	3 (42.9)	0.2
Number of graft arteries, *n* (%)	*n* = 1	55 (82.1)	46 (85.2)	5 (83.3)	4 (57.1)	0.4
	*n* = 2	11 (16.4)	7 (13.0)	1 (16.7)	3 (42.9)	
	*n* = 3	1 (1.5)	1 (1.9)	0 (0)	0 (0)	
Graft with multiple veins, *n* (%)		0 (0)	0 (0)	0 (0)	0 (0)	–
*Kidney procurement outcomes*						
Cold ischemia time (h), median (IQR)		14.8 (11.0–19.0)	14.8 (11.8–18.3)	12.3 (6.8–21.3)	18.0 (11.0–19.0)	0.8
Warm ischemia time in cDCD and uDCD (min), median (IQR)		20.0 (8.3–42.0)	–	16.0 (4.8–19.8)	40.0 (8.3–58.3)	0.2
Organ preservation solution, *n* (%)	Celsior	40 (59.7)	32 (59.3)	1 (16.7)	7 (100)	**0.02**
	UW	8 (11.9)	7 (13.0)	1 (16.7)	0 (0)	
	Custodiol	11 (16.4)	10 (18.5)	1 (16.7)	0 (0)	
	Unknown	8 (11.9)	5 (9.3)	3 (50.0)	0 (0)	

BMI = body mass index; cDCD = donor after controlled circulatory death; DBD = donor after brain death; eGFR = estimated glomerular filtration rate; GN = glomerulonephritis; IGA = immunoglobulin A; IQR = interquartile range; uDCD = donor after uncontrolled circulatory death.

The main type of donors was DBDs (80.6% [54/67]) followed by uDCDs (10.4% [7/67]) and cDCDs (9.0% [6/67]). The causes of death were different between the groups: stroke was the main cause of death in the DBD and cDCD groups, and cardiac arrest was the main cause in the uDCD group (*p* < 0.0001). The median donor age was 54.0 yr (IQR 39.5–62.5) and 49% were male. The median recipient age was 36.0 yr (IQR 24.0–58.0) and 64% were male. The median recipient body mass index was 24.8 kg/m^2^ (IQR 23.0–27.8). The majority was right-sided graft (55%), and 12 transplants (18%) had multiple renal arteries.

The median cold ischemia time was 14.8 h (IQR 11.0–19.0). The median warm ischemia time was 16.0 min (IQR 4.8–19.9) in the cDCD group and 40.0 min (IQR 8.3–58.3) in the uDCD group.

### 3.2 Intraoperative outcomes

Eight patients (11.9%) had orthotopic DD-RAKT and 59 patients (88.1%) had heterotopic DD-RAKT (Table 2). The primary indication of orthotopic DD-RAKT was the presence of severe vasculopathy and calcification at the level of aortoiliac vessels. Fourteen DD-RAKT procedures were performed in the left iliac fossa. The median operative time and the median rewarming time were 220.0 min (IQR 180.0–252.0) and 50.0 min (IQR 42.0–60.0) respectively (Table 2). Overall, seven (10.5%) intraoperative complications occurred, including six (9.0%) major intraoperative complications (three cases of active bleeding during the procedure, two cases of graft nephrectomy due to thrombosis during the procedure, and one case of open surgical conversion due to difficulties in performing the ureterovesical anastomosis), according to the EAUiaiC (Table 2).

**Table 2:** Intraoperative outcomes of deceased donor RAKT

Intraoperative outcomes		Overall population (*n* = 67)	DBD (*n* = 54)	cDCD (*n* = 6)	uDCD (*n* = 7)	*p* value
Transplant site, *n* (%)	Orthotopic	8 (11.9)	6 (11.1)	2 (33.3)	0 (0)	0.2
	Heterotopic	59 (88.1)	48 (88.9)	4 (66.7)	7 (100)	
Intraoperative complications, *n* (%)	EAUiaiC grade 0	0 (0)	0 (0)	0 (0)	0 (0)	0.4
	EAUiaiC grade 1	0 (0)	0 (0)	0 (0)	0 (0)	
	EAUiaiC grade 2 Venous thrombosis requiring a new venous anastomosis	1 (1.5)	1 (1.9)	0 (0)	0 (0)	
	EAUiaiC grade 3 Active bleeding	3 (4.5)	3 (5.6)	0 (0)	0 (0)	
	EAUiaiC grade 4A Transplant nephrectomy	2 (3.0)	1 (1.9)	0 (0)	1 (14.3)	
	EAUiaiC grade 4B Open surgical conversion to perform ureterovesical anastomosis	1 (1.5)	0 (1.9)	1 (16.7)	0 (0)	
	Total	7 (10.5)	5 (9.3)	1 (16.7)	1 (14.3)	
Surgical conversion, *n* (%)		2 (3.0)	1 (1.9)	1 (16.7)	0 (0)	0.1
Operative time (incision to closure; min), median (IQR)		220.0 (180.0–252.0)	200.0 (178.0–248.5)	272.5 (224.5–389.3)	240.0 (208.0–261.0)	0.1
Console time (min), median (IQR)		178.0 (138.0–205.0)	170.5 (130.0–201.3)	216.0 (152.8–310.0)	178.0 (159.0–205.0)	0.3
Arterial anastomosis time (min), median (IQR)		18.0 (14.0–22.0)	18.0 (13.5–21.5)	19.0 (16.5–25.0)	18.0 (17.0–27.0)	0.5
Venous anastomosis time (min), median (IQR)		20.0 (16.0–22.0)	19.0 (15.5–22.0)	16.0 (14.5–20.5)	22.0 (10.0–25.0)	0.1
Ureterovesical anastomosis time (min), median (IQR)		17.0 (13.0–24.5)	16.0 (13.0–25.0)	21.0 (17.0–23.5)	15.0 (10.5–26.5)	0.5
Rewarming time (min), median (IQR)		50.0 (42.0–60.0)	49.5 (42.0–60.0)	43.5 (39.3–52.3)	61.0 (56.0–62.0)	0.1

cDCD = donor after controlled circulatory death; DBD = donor after brain death; EAUiaiC = Intraoperative Adverse Incident Classification by the European Association of Urology; IQR = interquartile range; RAKT = robotic-assisted kidney transplantation; uDCD = donor after uncontrolled circulatory death.

### 3.3 Early postoperative and functional outcomes

The description and Clavien-Dindo grade of the different complications are reported in Table 3. Major postoperative surgical complications (Clavien-Dindo ≥3) occurred in 11 patients (16%), without differences between groups. Four graft nephrectomies were performed due to vascular thrombosis, three patients underwent endoscopic surgical procedure due to urinary stenosis or fistula, and two patients underwent an angioplasty and stenting of the arterial anastomosis due to transplant renal artery stenosis. One patient died due to active bleeding during the surgical procedure. The median length of hospitalization was higher in the uDCD group (12.5 vs 11.5 vs 33.0 d in the DBD vs cDCD vs uDCD group, *p* = 0.03)

**Table 3:** Early (POD 90) postoperative and functional outcomes of deceased donor RAKT

		Overall population (*n* = 67)	DBD (*n* = 54)	cDCD (*n* = 6)	uDCD (*n* = 7)	*p* value
*Early postoperative outcomes (POD 90)*						
Overall length of hospitalization (d), median (IQR)		13.0 (9.0–19.0)	12.5 (9.0–18.8)	11.5 (9.0–23.0)	33.0 (20.5–45.0)	**0.03**
Highest-grade postoperative surgical complications (according to Clavien-Dindo classification), *n* (%)	Grade 1	0 (0)	0 (0)	0 (0)	0 (0)	0.9
	Grade 2					
	Bleeding requiring transfusion	3 (4.5)	3 (5.6)	0 (0)	0 (0)	
	Tonic-clonic seizures	1 (1.5)	1 (1.9)	0 (0)	0 (0)	
	Lymphocele infection	2 (3.0)	2 (3.7)	0 (0)	0 (0)	
	UTI	4 (6.0)	3 (5.6)	0 (0)	1 (14.3)	
	Deep venous thrombosis	1 (1.5)	0 (0)	0 (0)	1 (14.3)	
	Paralytic ileus	2 (3.0)	1 (1.9)	1 (16.7)	0 (0)	
	Grade 3a	0 (0)	0 (0)	0 (0)	0 (0)	
	Grade 3b					
	Graft nephrectomy (thrombosis)	4 (6.0)	3 (5.6)	0 (0)	1 (14.3)	
	Laparoscopic marsupialization	1 (1.5)	1 (1.9)	0 (0)	0 (0)	
	Endoscopic reintervention (ureterovesical anastomosis stenosis or fistula)	3 (4.5)	2 (3.7)	1 (16.7)	0 (0)	
	Transplant renal artery stenosis requiring angioplasty + stenting	2 (3.0)	2 (3.7)	0 (0)	0 (0)	
	Grade 4a	0 (0)	0 (0)	0 (0)	0 (0)	
	Grade 4b	0 (0)	0 (0)	0 (0)	0 (0)	
	Grade 5	1 (1.5)	1 (1.9)	0 (0)	0 (0)	
Major postoperative surgical complication (Clavien-Dindo grade ≥3), *n* (%)		11 (16.4)	9 (16.7)	1 (16.7)	1 (14.3)	0.9
*Early functional outcomes (POD 90)*						
Delayed graft function, *n* (%)		18 (26.9)	14 (25.9)	1 (16.7)	3 (42.9)	0.6
eGFR (ml/min/1.73 m^2^), median (IQR)	POD 7	23.0 (8.7–41.0)	29.3 (10.7–43.0)	16.0 (6.0–29.0)	8.4 (7.0–13.0)	**0.03**
	Hospital discharge	38.0 (22.0–51.0)	38.0 (24.0–52.3)	29.0 (22.0–40.0)	21.0 (8.4–42.0)	0.4
Trifecta, *n* (%)		34 (50.7)	33 (61.1)	0 (0)	1 (14.3)	**0.001**

cDCD = donor after controlled circulatory death; DBD = donor after brain death; eGFR = estimated glomerular filtration rate; IQR = interquartile range; POD = postoperative day; RAKT = robotic-assisted kidney transplantation; uDCD = donor after uncontrolled circulatory death; UTI = urinary tract infection.

Eighteen patients (27%) experienced DGF, without differences between groups. The median eGFR at hospital discharge was 38.0 ml/min/1.73 m^2^. The trifecta was achieved by 51% of patients and mainly in the DBD group (*p* = 0.001; Table 3).

### 3.4 Follow-up outcomes

**Fig. 3:**
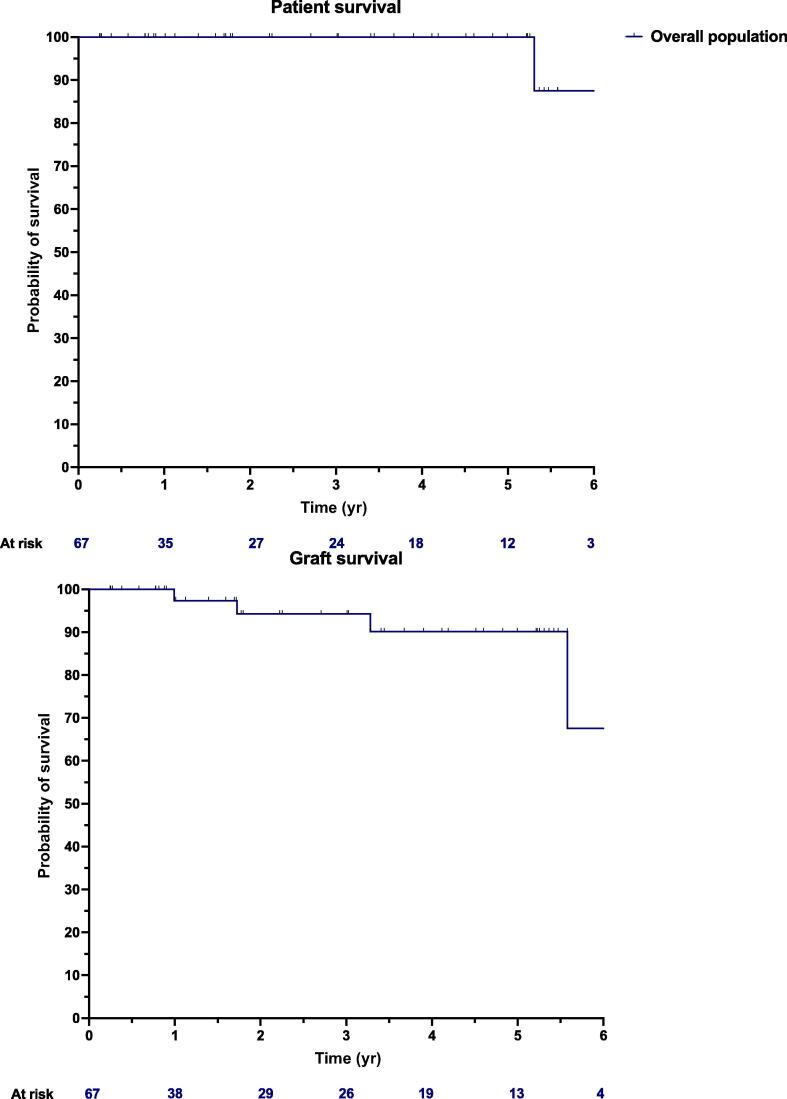
Six-year patient and graft survival in deceased donor robotic-assisted kidney transplantation cohorts.

The median follow-up was 27.1 mo (IQR 9.5–59.4; Table 4). The patient and graft survival are shown in Fig. 3. The last median eGFR was 52.4 ml/min/1.73 m^2^ (IQR 32.5–68.8; Table 4). No graft nephrectomy or KT-related surgical reinterventions occurred during follow-up.

**Table 4:** Follow-up outcomes of deceased donor RAKT

Follow-up outcomes	Overall population (*n* = 67)	DBD (*n* = 54)	cDCD (*n* = 6)	uDCD (*n* = 7)	*p* value
Follow-up (mo), median (IQR)	27.1 (9.5–59.4)	21.9 (9.5–53.5)	41.5 (10.7–64.6)	60.8 (33.0–67.9)	0.1
Graft nephrectomy after POD 90, *n* (%)	0 (0)	0 (0)	0 (0)	0 (0)	–
KT-related surgical reinterventions after POD 90, *n* (%)	0 (0)	0 (0)	0 (0)	0 (0)	–
Patient survival at last follow-up	97.0% (65/67)	98.1% (53/54)	83.3% (5/6)	100% (7/7)	0.1
Graft survival at last follow-up	92.6% (62/67)	92.6% (50/54)	83.3% (5/6)	100% (7/7)	0.4
eGFR at last follow-up (ml/min/1.73 m^2^), median (IQR)	55.0 (41.5–70.0)	53.8 (34.0–69.0)	55.0 (49.0–58.0)	63.6 (47.0–72.4)	0.9

cDCD = donor after controlled circulatory death; DBD = donor after brain death; eGFR = estimated glomerular filtration rate; IQR = interquartile range; KT = kidney transplantation; POD = postoperative day; RAKT = robotic-assisted kidney transplantation; uDCD = donor after uncontrolled circulatory death.

## 4 Discussion

During the past decade, minimally invasive surgery has been implemented progressively in KT to mitigate the drawbacks of open surgery [5,10,26,27], starting from the adoption of a pure laparoscopic approach. Following the spread of robotic platforms since the early 2000s [28], RAKT has been standardized progressively and implemented in some expert centers, aiming to reduce the morbidity of KT [[4], [5], [6], [7],^10^,^11^,^29^].

Currently, the major available evidence of the benefits of the minimally invasive surgery is based on studies evaluating the results of RAKT from living donors and in elective conditions. Therefore, few studies evaluated the safety and outcomes of RAKT in the complex situation of DDs, in emergency conditions, which is nevertheless the main source of transplants worldwide.

To the best of our knowledge, this study is the first multicentric prospective study reporting the intraoperative, perioperative, and midterm functional outcomes of DD-RAKT. Thus, despite higher logistical complexity, this study confirms that RAKT can be applied in the setting of deceased kidney donation, in carefully sectioned recipients by highly experienced surgeons. We reported a major postoperative surgical complication rate of 16.4% (comparable with the major postoperative surgical complications reported in the literature [16,18,19]). We also reported an optimal graft and patient survival at 27.1 mo of follow-up, while nearly 20% were DCDs and 39% were expanded criteria donors. Similarly, the reported DGF rate was comparable with the rates reported in the literature (21.7% for the DBD cases, 40% for the DCD cases with warm ischemia time ≤30 min, and 60% for the DCD cases with warm ischemia time >30 min) [30]. The OPTN/SRTR 2022 Annual Data Report on Kidney Transplantation confirmed our results: the reported DGF rate was 26.3%. The 5-yr graft survival rate was 81.4% for recipients aged 18–34 yr and 67.8% for recipients aged 65 yr or older. Thus, our results confirm the efficacy and safety of DD-RAKT in selected recipients.

In the setting of DDs, RAKT requires a well-structured decision-making process aiming to optimize organization of the surgical procedure. The decision-making process for the selection of RAKT candidates from DDs was similar in all the centers included, with the aim of reducing the duration of cold ischemia as much as possible. In addition, in order to obtain the best possible outcomes, the duration of warm ischemia for transplants from DCDs was limited to 60 min [31].

Previous studies have demonstrated the feasibility and safety of RAKT in the context of DDs. Campi et al [18] compared the outcomes of 21 DD-RAKT and 117 DD–OKT cases. They reported similar postoperative complications rates, eGFR trajectories, and patient and graft survival between the groups. The DGF rate was higher in the OKT cohorts. Spaggiari et al [19] reported the University of Illinois at Chicago experience on DD-RAKT. They performed 93 DD-RAKT procedures between 2009 and 2021. They reported a higher DGF rate in the obese population. Death-censored graft survival at 5 yr was significantly better than that in the UNOS-matched OKT cohort. Thus, our study reinforces that the use of the robotic approach is safe in the context of DDs.

Although midterm outcomes are reported in this study, the long-term safety of RAKT has recently been reported by Territo et al [29]. In this multicenter cohort study, intra- and postoperative complication rates were low, while long-term patient (98.9%) and graft survival (98.2%) rates were optimal. In this study, rewarming time (43 min) and total operative time (210 min) were similar to those reported in our study.

### 4.1 Study limitations

The present study is not devoid of limitations. First, this study is a retrospective analysis of a prospectively maintained database from seven European centers; as such, the risks of selection bias, detection bias, and residual confounding cannot be ruled out. Second, the surgical technique for DD-RAKT was not standardized in all centers, with specific nuances added by each team (despite adherence to the principles of the ERUS technique for RAKT from living donors) [29]. Third, only midterm and postoperative outcomes of DD-RAKT were evaluated. Fourth, no comparison with the traditional open approach was performed. Finally, RAKT was performed at high-volume referral centers by highly trained transplant teams. Therefore, our findings might not be generalizable to all clinical scenarios.

## 5 Conclusions

DD-RAKT is feasible and safe at experienced referral European centers, providing a viable way of expanding transplantation access to patients with ESKD. Notwithstanding the logistical barriers for DD-RAKT, careful recipient selection and organ matching are key factors to achieve the best perioperative and functional outcomes.

  ***Author contributions*:** Thomas Prudhomme had full access to all the data in the study and takes responsibility for the integrity of the data and the accuracy of the data analysis.

  *Study concept and design*: Prudhomme, Peri, Pecoraro, Musquera, Campi.

*Acquisition of data*: Prudhomme, Peri, Pecoraro, Territo, Etcheverry, Ortved, Røder, Mirza, Vignolini, Rohrsted, de Mesa Rodriguez, Mercier, Doumerc, Masieri, Vigues, Breda, Serni, Alcaraz, Musquera, Campi.

*Analysis and interpretation of data*: Prudhomme, Peri, Pecoraro, Territo, Etcheverry, Ortved, Røder, Mirza, Vignolini, Rohrsted, de Mesa Rodriguez, Mercier, Doumerc, Masieri, Vigues, Breda, Serni, Alcaraz, Musquera, Campi.

*Drafting of the manuscript*: Prudhomme, Peri, Pecoraro.

*Critical revision of the manuscript for important intellectual content*: Musquera, Campi.

*Statistical analysis*: Prudhomme, Pecoraro, Campi.

*Obtaining funding*: Prudhomme.

*Administrative, technical, or material support*: None.

*Supervision*: Musquera, Campi.

*Other*: None.

  ***Financial disclosures:*** Thomas Prudhomme certifies that all conflicts of interest, including specific financial interests and relationships and affiliations relevant to the subject matter or materials discussed in the manuscript (eg, employment/affiliation, grants or funding, consultancies, honoraria, stock ownership or options, expert testimony, royalties, or patents filed, received, or pending), are the following: None.

  ***Funding/Support and role of the sponsor*:** Thomas Prudhomme received a grant from the European Urological Scholarship Programme (EUSP) and the French Association of Urology.
